# Soft ankle exoskeleton to counteract dropfoot and excessive inversion

**DOI:** 10.3389/fnbot.2024.1372763

**Published:** 2024-08-21

**Authors:** Xiaochen Zhang, Yi-Xing Liu, Ruoli Wang, Elena M. Gutierrez-Farewik

**Affiliations:** ^1^KTH MoveAbility, Department of Engineering Mechanics, KTH Royal Institute of Technology, Stockholm, Sweden; ^2^Department of Women's and Children's Health, Karolinska Institutet, Stockholm, Sweden

**Keywords:** assistive device, biomechanics, gait impairment, gait analysis, soft robotics

## Abstract

**Introduction:**

Wearable exoskeletons are emerging technologies for providing movement assistance and rehabilitation for people with motor disorders. In this study, we focus on the specific gait pathology dropfoot, which is common after a stroke. Dropfoot makes it difficult to achieve foot clearance during swing and heel contact at early stance and often necessitates compensatory movements.

**Methods:**

We developed a soft ankle exoskeleton consisting of actuation and transmission systems to assist two degrees of freedom simultaneously: dorsiflexion and eversion, then performed several proof-of-concept experiments on non-disabled persons. The actuation system consists of two motors worn on a waist belt. The transmission system provides assistive force to the medial and lateral sides of the forefoot via Bowden cables. The coupling design enables variable assistance of dorsiflexion and inversion at the same time, and a force-free controller is proposed to compensate for device resistance. We first evaluated the performance of the exoskeleton in three seated movement tests: assisting dorsiflexion and eversion, controlling plantarflexion, and compensating for device resistance, then during walking tests. In all proof-of-concept experiments, dropfoot tendency was simulated by fastening a weight to the shoe over the lateral forefoot.

**Results:**

In the first two seated tests, errors between the target and the achieved ankle joint angles in two planes were low; errors of <1.5° were achieved in assisting dorsiflexion and/or controlling plantarflexion and of <1.4° in assisting ankle eversion. The force-free controller in test three significantly compensated for the device resistance during ankle joint plantarflexion. In the gait tests, the exoskeleton was able to normalize ankle joint and foot segment kinematics, specifically foot inclination angle and ankle inversion angle at initial contact and ankle angle and clearance height during swing.

**Discussion:**

Our findings support the feasibility of the new ankle exoskeleton design in assisting two degrees of freedom at the ankle simultaneously and show its potential to assist people with dropfoot and excessive inversion.

## 1 Introduction

Dropfoot, or the inability to lift the foot during gait, is a common secondary gait disorder after a stroke (Kluding et al., 2013) or other neurological injuries (Nori and Das, 2019) resulting from weakness and/or atypical motor control. People with dropfoot often exhibit two gait pathologies (Blaya and Herr, 2004): steppage gait and excessive subtalar inversion. Steppage gait is a condition where people demonstrate the inability to lift the forefoot during the swing phase adequately and initially contact the floor with the whole foot or forefoot (Perry and Burnfield, 2010), making a high risk of tripping or even falling; excessive subtalar inversion after a stroke is primarily caused by spasticity in tibialis posterior. It can further influence foot clearance, leading to an unstable base of support and a high risk of ankle injury during walking (DeMers et al., 2017), and is associated with gait asymmetry and slow walking speed (Deltombe et al., 2017; Li, 2020). In dropfoot gait, typical compensatory movements to achieve foot clearance include ipsilateral pelvic elevation or “hiking” and increased hip abduction or “circumduction.” Together, these movements generally reduce walking efficiency and endurance (Schmid et al., 2013), influence walking independence (Awad et al., 2017) and confidence (Yeung et al., 2021), and generally make daily activities challenging and inconvenient (Gil-Castillo et al., 2020).

Orthotic devices, such as ankle-foot orthoses that compensate for dropfoot gait by restricting plantarflexion, are widely used and positively impact the mobility and balance of the user (Tyson and Kent, 2013; Winstein et al., 2016; Awad et al., 2017). However, orthoses that restrict ankle movement inhibit any remaining ability of the plantarflexors to forward propel the leg during the preswing phase (Vistamehr et al., 2014) and may induce dependence on the devices (de Sèze et al., 2011; Daryabor et al., 2018).

Powered wearable exoskeletons are with increasing frequency being developed for movement assistance and rehabilitation (Blaya and Herr, 2004; Wu et al., 2019; Hu et al., 2023; Zhang L. et al., 2023). Rigid powered ankle exoskeletons may be able to provide adequate and timely assistance with movements, showing better performance than passive orthotic devices (Shorter et al., 2011; Yeung et al., 2018; Kim et al., 2020). The implementation of an actuation and control systems may achieve this, but rigid structures tend to be heavy and bulky, and may thus have limited feasibility and efficacy (Liu et al., 2021). It is also challenging to customize rigid exoskeletons for individual users as alignment of the device's ankle joint with the user's ankle joint can be challenging.

To this end, exoskeletons are increasingly designed to be lighter and more compliant, with the aim of achieving natural interactions between them and their users (Bae et al., 2015; Thalman et al., 2019). Many soft exoskeletons consist of an actuation system and a cable transmission system with a compact size and high transmission efficiency (Bae et al., 2015). The actuation system, which is often the heaviest part, should be positioned near the body's center of mass instead of distally near the ankle to minimize the user's additional metabolic demand associated with its weight (Browning et al., 2007; Lerner et al., 2018). Ankle exoskeletons with cable-driven transmission can have a substantial effect on both plantarflexion and dorsiflexion for people with disabilities, showing potential to improve gait kinematics (Bae et al., 2018), mobility, and energy expenditure (Lerner et al., 2018; Han et al., 2021). Nowadays, most soft ankle exoskeletons have focused solely on assisting the ankle in the sagittal plane (Bae et al., 2018; Lerner et al., 2018), providing assistance with toe clearance and push-off to fulfill the basic requirements for walking. However, they often overlook the critical need for assistance in the frontal plane, which is also vital for maintaining foot clearance and landing stability, thus impacting gait safety, especially in cases of excessive inversion. Although few attempts have been made to address multi-degree-of-freedom (DoF) requirements, these solutions typically involve complex structures and control strategies (Park et al., 2014).

The aims of this study were therefore to design and fabricate a 2-DoF powered soft ankle exoskeleton specifically intended to assist both dropfoot and excessive inversion, with features of lightweight, natural interaction, and reasonably simple mechanical design, and, in non-disabled subjects with simulated dropfoot impairment, to test its feasibility in assisting dorsiflexion and subtalar eversion, resisting plantarflexion, and compensating for device resistance in seated subjects, and in improving ankle and foot kinematics during gait.

## 2 Design

### 2.1 Hardware design

**Figure 1:**
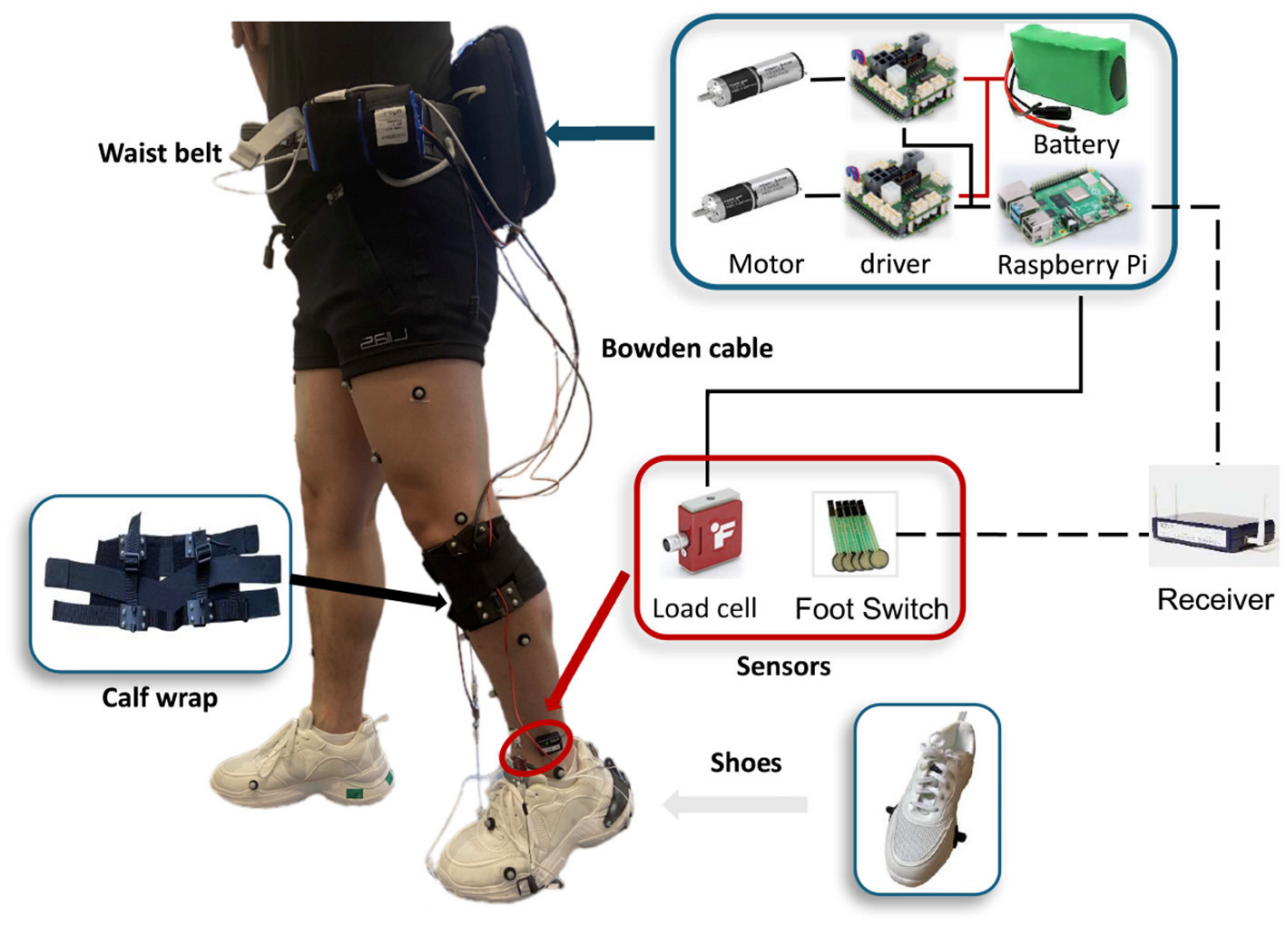
Overview of the soft ankle exoskeleton designed to counteract dropfoot and excessive inversion.

Inspired by the Harvard exosuits (Awad et al., 2017; Bae et al., 2018), the design of our active soft ankle exoskeleton consisted of four parts: a waist belt, a calf wrap, a pair of shoes, and sensors, with a total mass of 3.1 kg (Figure 1).

The design principles and selection of components were guided by the goal to develop a compliant and lightweight structure, with minimal burden on the user, maximal comfort, and flexibility to accommodate for different body sizes.

#### 2.1.1 Waist belt

The actuation module was attached to the waist belt, consisting of actuators, controllers, and power supplies.

The actuator module contains two motors. In each actuator, a brushless DC motor (EC-4pole 22, 90W, Maxon Inc, Switzerland) with a planetary gearbox (GP 32, HP 123:1, Maxon Inc, Switzerland) was used, which can provide up to 5.54 Nm torque. Through a 3D printed pulley with a radius of 14 mm, the inner Bowden cable was attached to the actuators, with a possible retraction force of up to 396 N, which fulfilled the application requirements in our design.

A microcomputer (Raspberry Pi 4B, Raspberry Pi Foundation, UK) was used to control the actuators by sending control signals to motor drivers (EPOS4 Compact 50/8 CAN, Maxon Inc, Switzerland). The microcomputer was monitored and controlled by a laptop via Virtual Network Computing, through which the controller settings and the exoskeleton parameters could be tuned and adjusted remotely. By processing the movement information extracted from the load sensors, the microcomputer sent the control signal to the motor drivers, and the actuator was then driven to follow the control profiles.

The power supply was comprised of two batteries (Li-ion)—one with a capacity of 24 V and 144 Wh and the other at 18V—as well as a power bank with an output of 5 V and 3 A, which powered the actuators, load cells, and microcomputer, respectively.

#### 2.1.2 Calf wrap

The calf wrap is made of Neoprene, secured with Velcro straps and elastic bands, and is able to fit different shank dimensions. Two anchors were assembled on the bottom of the calf wrap, positioned on both the medial and lateral sides, and were used to connect to the sheath of Bowden cables.

#### 2.1.3 Shoe

Anchors were fixed on the lateral and medial sides of the forefront of the shoes. The distal end of each of the two inner Bowden cables was attached to these anchors.

#### 2.1.4 Sensors

Two load cells (LSB205, FUTEK) with amplifiers (A100, FUTEK) were aligned in series with the Bowden cables to measure cable tensile forces, and the measured data were transferred to the controller through the cables. Encoders (16 EASY, 1024 CPT, 3 channels, Maxon Inc, Switzerland) mounted on motors were used to monitor motor positions. Two pairs of foot switches (Cometa, Italy) with four transducers each were attached to the bottom of shoes. The foot switch signal was transmitted from the receiver to the microcomputer via the TCP/IP protocol, at a frequency of 100 Hz. This signal was used to detect gait events, thereby identifying stance and swing phases.

The device was designed with both electrical and mechanical stops. For the electrical stop, the position of the cable and motor can be detected during the trials, and if the motor's position is out of a safe range, the motor will be stopped. In the mechanical stop, there is a physical stop in the cable to make sure it cannot be over-retracted.

### 2.2 Controller

**Figure 2:**
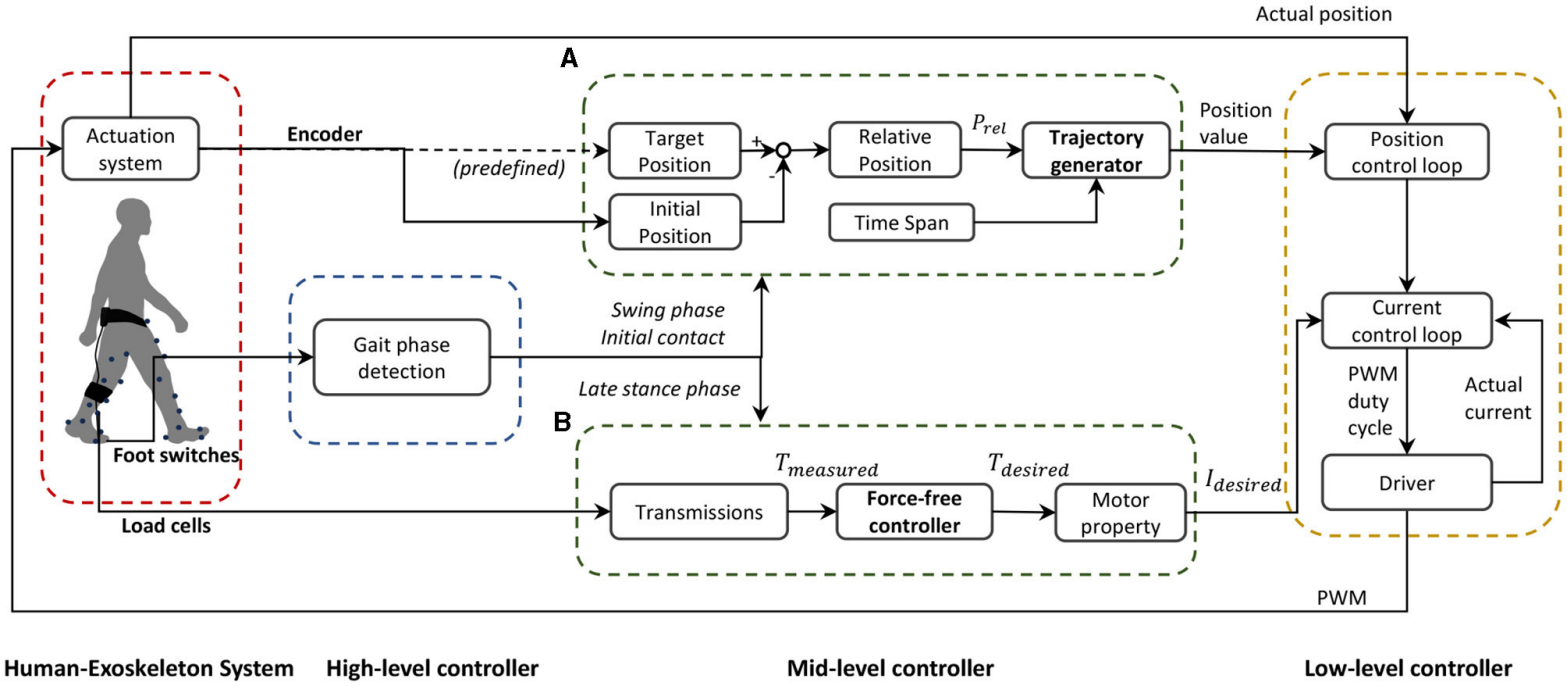
Schematic block diagram of the soft ankle exoskeleton controller. The frame presents a human-exoskeleton system, a high-level controller, a mid-level controller, and a low-level controller. The mid-level controller consists of two different modes: **(A)** position trajectory generator and **(B)** force-free controller.

A hierarchical controller that consists of a high-level, a mid-level, and a low-level controller was developed in this study (Figure 2). The high-level controller detects the gait phase based on foot switches on the bottom of the shoes. The mid-level controller has two modes—position control to achieve the desired ankle joint profile (Figure 2A) and force-free control to compensate for the resistance generated in the actuation system (Figure 2B). The low-level controller has a configuration with position control and current control loops embedded in the motor driver that aims to precisely direct the actuator to follow the profiles from the mid-level controller.

#### 2.2.1 Position trajectory generator

On the exoskeleton, one anchor located on the calf wrap, one on the shoe, and one along the ankle joint axis formed a triangular structure, on both medial and lateral sides. When the actuator is activated, the interior angles of the triangle undergo alterations followed by the cable retraction or release, driving the ankle joint moving in the sagittal plane. Different alterations can be realized in the two triangular structures by setting different profiles for the two motors, which then trigger the kinematic change of the ankle joint in the sagittal and frontal planes simultaneously.

**Figure 3:**
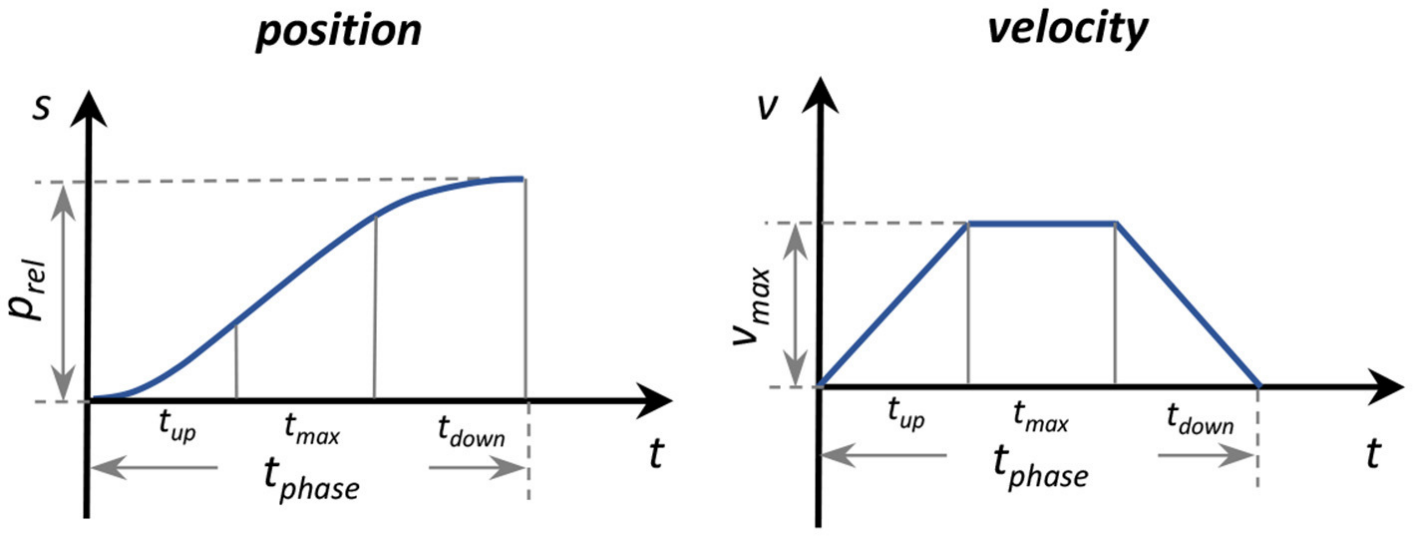
Parameterization of the position trajectory three-phase velocity profile.

We generated the position trajectory that characterized the desired ankle motion with a smooth property at the initial and end stages. The position trajectory was defined by relative position *p*_*rel*_ and phase time *t*_*phase*_, as well as a three-phase velocity profile (Figure 3). The function of the trajectory generator is to translate this predefined profile to the parameters that the low-level controller can read.

Relative position *p*_*rel*_ was obtained by subtracting initial position *p*_*ini*_ from target position *p*_*tar*_. The positions were measured as the motor shaft positions by the encoders. The phase time *t*_*phase*_ was predefined and divided into three equal phases: ramp up *t*_*up*_, maximum speed *t*_*max*_, and ramp down *t*_*down*_. With the relative position *p*_*rel*_ and specified intervals of *t*_*phase*_, maximum velocity *v*_*max*_, acceleration *a*_*max*_, and deceleration *a*_*min*_ were computed. The position trajectory was then determined accordingly (Figure 2A).

To ensure precise motor performance along the predefined trajectory, the motor driver used a nested control loop structure wherein the position controller generated a desired current command based on the error between the desired and actual positions. The current control loop then tracked this current command using a finely tuned PID controller.

#### 2.2.2 Force-free controller

A force-free controller, which aimed to compensate for the effect of gravity, device friction, and inertial forces (Dong et al., 2019; Hu et al., 2024), was used to achieve the desired ankle joint under a non-constraint condition during plantarflexion (Figure 2B).

In this study, the soft exoskeleton's weight and inertia around the ankle were considered negligible. The controller was simplified as follows:

The output torque of the motor *T*_*m*_ was computed using Equation 1:


(1) $$\begin{array}{l} T_{m}=\left(1-K_{t}\right)\cdot T \end{array}$$

where *T*_*m*_ and *T* represent the output torque of the motor and measured torque, respectively; *K*_*t*_ is a constant value.

Cable tensile force *F*_*cable*_ was measured with a load cell, and the measured torque was computed as per Equation 2:


(2) $$\begin{array}{l} T=F_{cable}\cdot R_{pulley}/r_{gear} \end{array}$$

where *R*_*pulley*_ is the radius of the pulley, and *r*_*gear*_ is the ratio of the gear box.

For the constant value *K*_*t*_, the relationship between the external torque, the constant value, and the inertia and friction torques can be expressed as per Equation 3:


(3) $$\begin{array}{l} T_{ext}=K_{t}^{-1}\left(J\ddot{\theta }+T_{f}\right) \end{array}$$

where *T*_*ext*_ is the external torque; $J\ddot{\theta }$ is the inertia torque of the motor rotor, and *T*_*f*_ is the friction torque.

It can be seen that if *K*_*t*_ > 1, the external torque that is needed to overcome the inertia and friction torques will decrease by a multiple of *K*_*t*_.

The current input *I* to the controller can be expressed as per Equation 4:


(4) $$\begin{array}{l} I=T_{m}/k_{\tau } \end{array}$$

where *k*_τ_ represents the torque constant of the motor.

After computing the current input, the low-level current control loop utilized a finely tuned PID controller to track the current input. By continuously adjusting based on the error between the actual and target current, the system maintained accurate motor operation (Figure 2). This ensured the motor was driven to achieve the desired performance in the force-free application.

## 3 Experiments

Proof-of-concept experiments were conducted in two sessions and with different non-disabled subjects. The first three tests were performed on subjects in a seated position, and the fourth test was performed on subjects walking in an instrumented gait laboratory. These experiments were conducted to test the exoskeleton's feasibility and basic functionality to:

1. Achieve two-DoF assistance simultaneously, specifically ankle dorsiflexion and eversion, from an initially plantarflexed and inverted ankle position.

2. Control speed and final position during a passive plantarflexion motion.

3. Compensate for device resistance from cable release during plantarflexion movement.

4. Normalize foot and ankle kinematics during walking.

### 3.1 Participants and experimental setup

Five non-disabled subjects (2M / 3F, (Mean ± SD) height: 166.4 ± 6.2 cm, weight: 60.6 ± 7.9 kg, age: 28.4 ± 1.4 years) participated in the seated tests, and three non-disabled subjects (1M / 2F, (Mean ± SD) height: 166.3 ± 5.8 cm, weight: 57.5 ± 3.9 kg, age: 27.4 ± 0.6 years) participated in the gait text. Inclusion criteria were no musculoskeletal disorders or recent lower-limb injuries that can influence gait or ankle movement. All subjects provided written informed consent, and the experiment was approved by the Swedish Ethical Review Authority (Dnr. 2023-02891-01).

Experiments were conducted in the Promobilia MoveAbility Lab, equipped with a 10-camera motion capture system (Vicon V16, UK). Thirty-six reflective markers were placed on each subject, and joint angles were computed according to a common lower-limb marker set (CGM2.4). Marker data were collected at 100 Hz. Two surface electromyography (EMG) sensors (Aktos Nano, Myon, Schwarzenberg, Switzerland) were placed on each subject's tibialis anterior (TA) and peroneus longus (PL) according to SENIAM recommendations (Hermens et al., 1999). Maximum voluntary isometric contraction (MVIC) of the two muscles was measured as per (Konrad, 2005), with the subject in a supine position on an examination table with heel contact.

In the first session, Tests 1–3, each subject, donning the exoskeleton and a 1-kg weight secured to the show over the lateral forefoot, sat on a chair with the shank and foot suspended in the sitting test. The purpose of the weight was to create an external plantarflexion and inversion moment, as a proxy to simulate dropfoot and excessive inversion impairments. The order of the three tests was randomized for each subject.

In the second session, Test 4, each subject donned the exoskeleton and the 1-kg weight over the lateral forefoot and walked on level ground at their preferred speed in a gait laboratory.

### 3.2 Test 1: assisting ankle dorsiflexion and eversion

This test focused on the functionality of assisting the ankle joint to dorsiflex and evert to a target position from an initially plantarflexed and inverted position. This test approximates dropfoot during the swing phase.

Prior to the data collection in Test 1, the target position was defined as the neutral position of the ankle during standing, and the initial position was defined from an earlier trial with wedges placed under the foot that placed the ankle in ~18° plantarflexion and 10° inversion. Just prior to data collection, the ankle was moved to the initial position, confirmed via 3D motion capture, while suspended. Motor positions were recorded at both ankle joint position setups by the encoders.

During the test, position control was used to assist ankle dorsiflexion and eversion of the ankle joint from the initial to the target position during two different time intervals—fast: 0.5 s and slow: 0.75 s. These were repeated three times in a randomized order.

### 3.3 Test 2: controlling plantarflexion

This test focused on efficacy in controlling and preventing excessive plantarflexion and inversion, approximating loading response in early stance, beginning at initial contact and ending at a plantigrade foot position. This test involved controlling a passive plantarflexion movement. Subjects were seated with feet suspended and the ankle initially in a neutral position. The 1-kg weight on the lateral forefoot then passively plantarflexed and inverted the ankle. The exoskeleton's objective was to control this motion to a target position in a predetermined duration.

The initial position was defined as the neutral position during standing, and the target position was set to 8° plantarflexion.

During the test, position control was used to control ankle plantarflexion by following the predefined cable release trajectory, determined by initial and target positions, during a 0.4 s duration (“with exo” mode). As a reference, subjects were also tested in this procedure with the exoskeleton, while the cables were detached (“no cables” mode).

With the exoskeleton donned, subjects were asked to relax and not to not perform any active dorsi- or plantarflexion. However, in the “no cables” mode, they were required to activate dorsiflexors prior to the test to hold up the foot to the neutral position and then asked to relax them to initiate the test.

The trials were each repeated three times, and the order of the two modes was randomized.

### 3.4 Test 3: resistance compensation during plantarflexion

This test focused on evaluating the efficacy of the force-free controller to compensate for device resistance during plantarflexion motion, approximating the preswing phase of gait. The exoskeleton's objective was to restrict desired plantarflexion as little as possible.

The initial position was defined as the neutral ankle position during standing. The 1-kg weight was placed on the lateral forefoot, and each subject passively planterflexed the ankle from a neutral position during three different conditions:

- The cables were detached from exoskeleton to measure natural plantarflexion movement without any resistance (“no cables” mode),
- The exoskeleton was unpowered, and the cables were stretched passively to measure the effects of resistance (“passive” mode),
- The exoskeleton was powered with the force-free controller, aiming to compensate for resistance and restore natural plantarflexion motion (“resistance compensation” mode).

Subjects were asked to be as relaxed as possible during the trials, so that plantarflexion was induced by gravity and the weight. As in Test 2, however, in the “no cables” mode, subjects were required to use dorsiflexor muscles to maintain ankle position prior to the test and then to relax them to initiate the test.

Each mode was repeated three times in a randomized order.

### 3.5 Test 4: assisting ankle and foot kinematics during gait

This test focused on the exoskeleton's efficacy in correcting the altered ankle and foot kinematics associated with the simulated dropfoot, specifically its ability to resist the passive plantarflexion and inversion from the simulated impairment, while not limiting the plantarflexion movement during stance.

Subjects walked on level ground at their preferred walking speeds in three different conditions:

- Walking without the exoskeleton and without the weight on the shoe. Parameters measured in this condition were set as reference (“Reference”).
- Walking without the exoskeleton and with the weight on the shoe. The simulated impairments were measured in this condition (“Simulated Impairment”).
- Walking with the powered exoskeleton (“With Exoskeleton”) and the weight on the shoe. Position control was used in swing and loading response phases, and the force-free controller was used in late stance. In the swing phase, the target position and phase time of the position control were defined as the neutral position during standing, similar to Test 1, and the average swing phase duration during the previous three steps. In loading response, the parameters were set to 8° plantarflexion and 0.2 s, respectively.

The order of conditions was randomized.

### 3.6 Data processing and outcome parameters

Recorded raw EMG signals were processed using a band-pass filter (Butterworth 20–400 Hz), rectified, low-pass filter (Butterworth 4 Hz), and then normalized by MVICs (MATLAB R2020b, Mathworks, US). Kinematics were calculated through inverse kinematics (Nexus, Vicon).

The device's functionality in the different seated tests was evaluated with three outcome parameters:

- Tracking accuracy, defined as the error between measured and target ankle angles in the sagittal and frontal planes, was computed for Tests 1 and 2.
- Resistance reduction, defined as the rate of ankle plantarflexion and inversion, was computed for each of the three modes—resistance compensation, passive, and no cables.
- Muscle activity, specifically the normalized EMG signals with and without resistance compensation for each subject, was computed.

Paired t-tests were used to compare velocities and normalized EMG in different modes in Test 3.

The devices' functionality to normalize foot and ankle kinematics during gait was evaluated with five outcome parameters:

- Foot inclination angle at initial contact
- Ankle inversion angle at initial contact
- Foot clearance height during the swing phase, defined as the minimal height of the fifth metatarsal head marker
- Maximum plantarflexion angle in preswing, defined as the maximum plantarlexion angle between 50 and 60 % gait cycle
- Average ankle angle in late swing, defined as the average sagittal ankle angle in 90%–100 % gait cycle.

No statistical tests were performed in Test 4, since there they only include data on three subjects.

## 4 Results

### 4.1 Test 1: performance in assisting dorsiflexion and eversion

**Figure 4:**
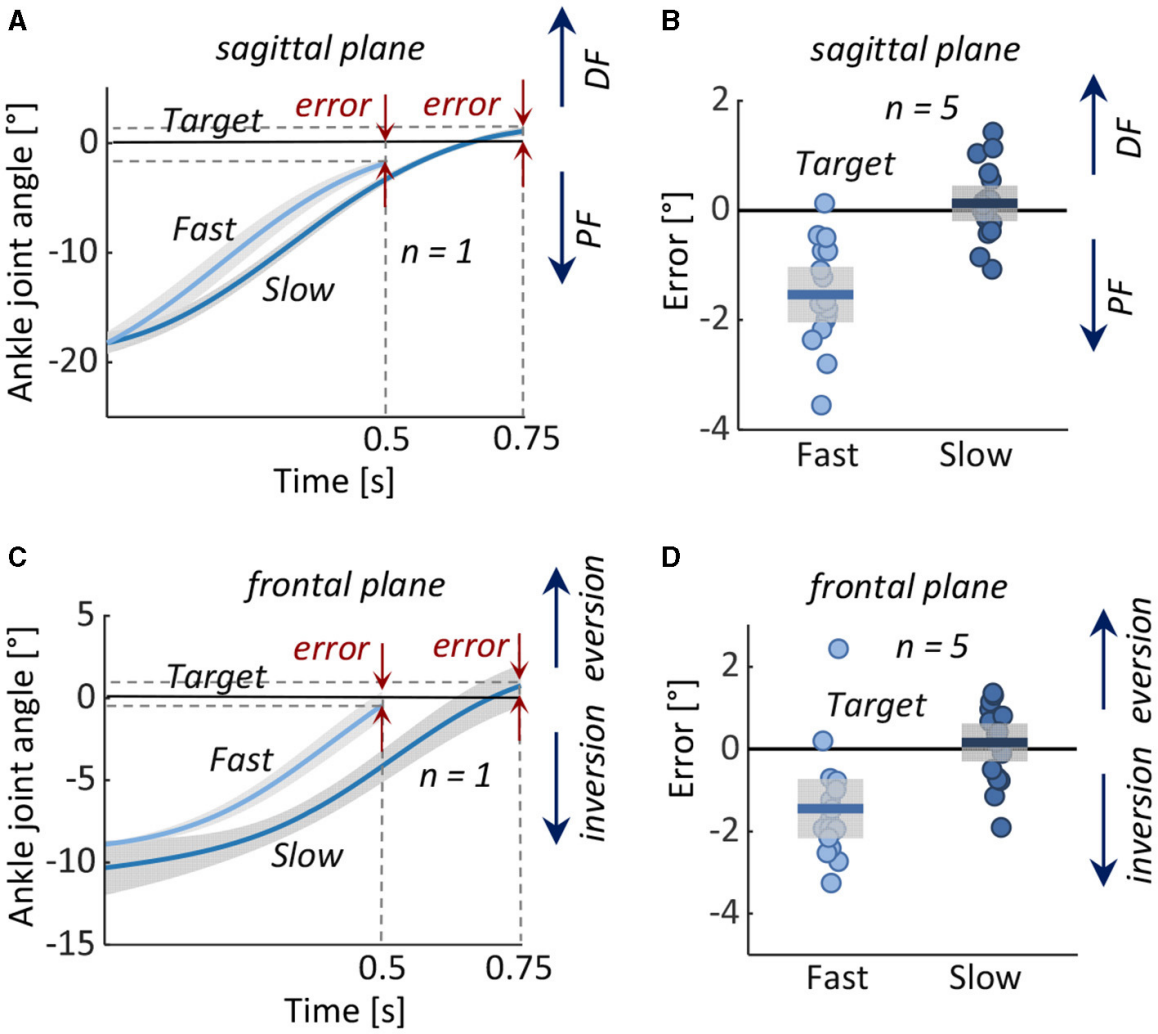
Test 1: Assisting dorsiflexion and eversion from an initial position of ankle plantarflexion and inversion to a neutral target position in 0.5 s (Fast) and 0.75 s (Slow). Left: Measured ankle joint angles (mean ± 1 standard deviation) during fast and slow movements in **(A)** representative subject in the **(A)** sagittal and **(C)** frontal planes. Right: Error in reaching the target angle in all five subjects in the **(B)** sagittal and **(D)** frontal planes. Boxes represent the standard deviation, the horizontal lines represent the mean value of the five subjects in the two conditions, and data from individual subjects are overlaid. DF, dorsiflexion; PF, plantarflexion.

The subjects' average initial ankle positions were ~18° plantarflexion and 10° inversion, and target positions were 0° in both planes. With the exoskeleton, the subjects were able to approximate the target position, in both fast and slow conditions (Figure 4). In both anatomical planes, slightly greater accuracy was achieved in the slow condition. Among subjects, the mean ± standard deviation (SD) of the target position in the sagittal plane were –1.5° ± 0.5° in the fast condition and 0.1° ± 0.3° in the slow condition and in the frontal plane, –1.4° ± 0.7° in the fast condition, and 0.2° ± 0.5° in slow condition, respectively.

### 4.2 Test 2: performance in controlling plantarflexion

**Figure 5:**
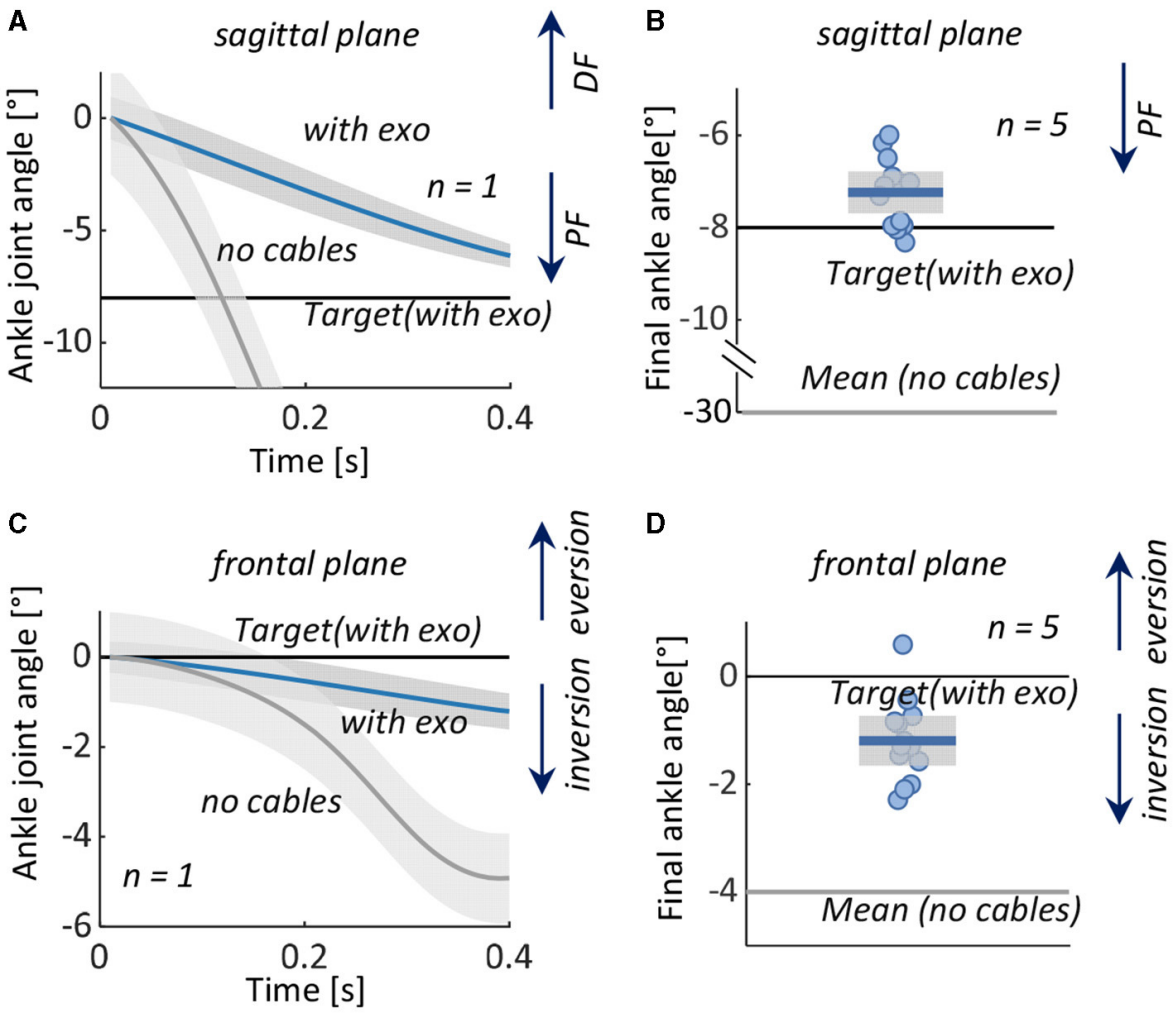
Test 2: Controlling plantarflexion from an initial neutral position to a plantarflexed position. Left: Measured ankle joint angles (mean ± 1 standard deviation) with the exoskeleton (with exo) and with cabled detached (no cables) in a representative subject in the **(A)** sagittal and **(C)** frontal planes. Right: Ankle angle at the final position in all five subjects and target angles in the **(B)** sagittal and **(D)** frontal planes. Boxes represent the standard deviation with data from individual subjects overlaid, the horizontal black lines represent the target angles of the five subjects with the exoskeleton, and the horizontal gray lines represent the mean value in the condition without the exoskeleton. DF, dorsiflexion; PF, plantarflexion.

In the no cables condition, the ankles rapidly and exaggeratedly plantarflexed and inverted. With the exoskeleton, ankle motion was decelerated and reduced in both sagittal and frontal planes (Figure 5).

Among subjects, with the exoskeleton, the mean ± SD ankle final angles were –7.2° ± 0.5° (with target position errors of 0.8° ± 0.5°) in the sagittal plane and –1.2° ± 0.5° (target position error 1.2° ± 0.5°) in the frontal plane. Without the exoskeleton, mean ± SD final angle was –30.8° ± 5.7° in the sagittal plane and –4.0° ± 1.7° in the frontal plane.

### 4.3 Test 3: performance in resistance compensation

**Figure 6:**
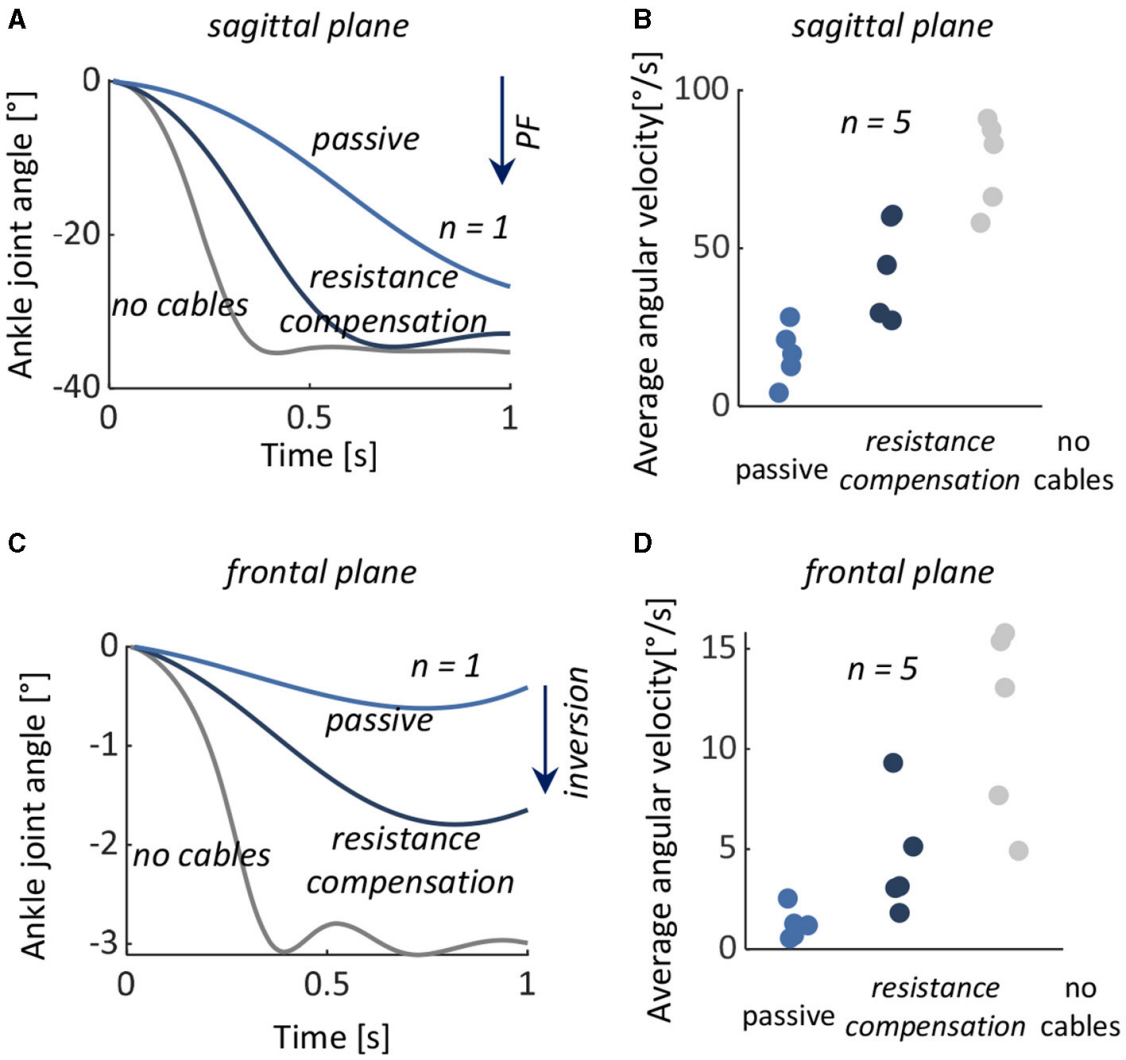
Test 3: Resistance compensation during a desired plantarflexion motion. Left: Measured ankle joint angles with the exoskeleton's natural resistance (passive), with the exoskeleton and force-free controller (resistance compensation), and with the cables detached from the exoskeleton (no cables) from a representative subject in the **(A)** sagittal and **(C)** frontal planes. Left: Average plantarflexion angular velocity in all five subjects during passive (light blue), resistance compensation (dark blue), and no cables (gray) in **(B)** sagittal and **(D)** frontal planes. The data from individual subjects are overlaid. PF, plantarflexion.

The device's natural resistance decelerated the desired plantarflexion motion. The resistance compensation was partially able to compensate for this resistance but not fully. The rate of ankle motion was lowest in the passive mode, with an average of 16.6 deg s^–1^ in the sagittal plane and 1.1 deg s^–1^ in the frontal plane. With the exoskeleton in force-free control mode, the average rate of ankle motion was significantly higher; it more than doubled in both the sagittal plane (44.4 deg s^–1^, *p* < 0.01) and in the frontal plane (4.4 deg s^–1^, *p* < 0.05). However, even with the force-free control, the ankle motion was approximately half as fast as in the no-cable mode (Figure 6).

There were no significant differences in muscle activities of the TA and PL during the trials between passive and force-free conditions (*p* = 0.618 and *p* = 0.836).

### 4.4 Test 4: performance in normalizing foot and ankle kinematics during gait

**Figure 7:**
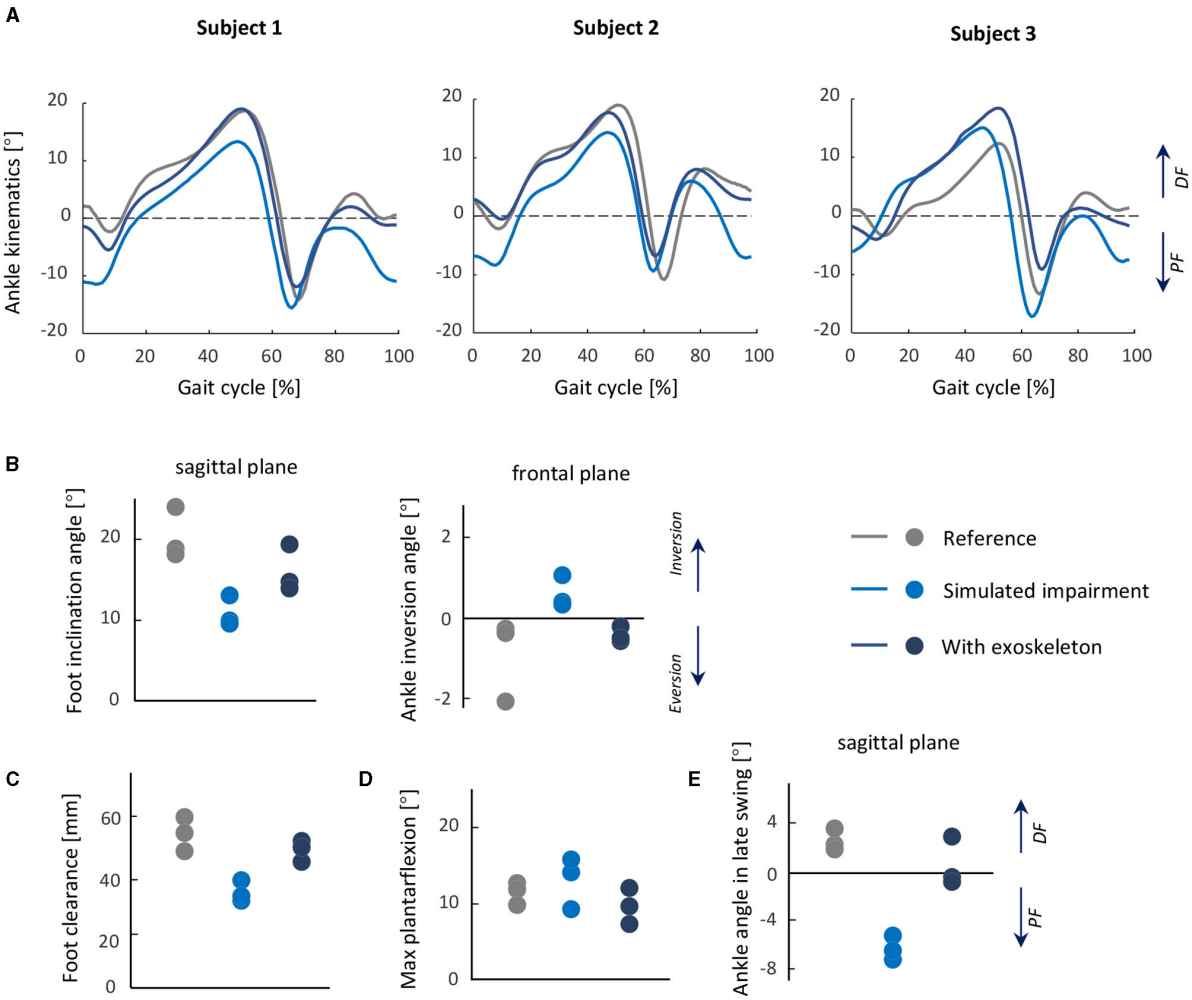
Test 4: Normalizing foot and ankle kinematics during gait from a simulated dropfoot condition. Top row: **(A)** Ankle joint kinematics during gait in each subject in three conditions: reference, simulated impairment, and with exoskeleton, shown as averages of each trial. **(B)** Foot inclination angle and ankle inversion angle at initial contact. **(C)** Foot clearance height during the swing phase. **(D)** Maximum plantarflexion during gait. **(E)** The average ankle joint angle in the late swing phase. From **(B)** to **(E)**, the mean value of each subject is shown for all metrics, and the data from individual subjects are overlaid.

Compared to the reference condition, the simulated impairment resulted in decreased ankle dorsiflexion in late swing and loading response, wherein the foot segment was both more plantarflexed and inverted at initial contact. The exoskeleton normalized these angles (Figure 7); with the exoskeleton, the average foot inclination angle at initial contact increased from 11.6° to 16.9° (reference 20.8°), and the ankle inversion decreased from 1.0° inversion to 0.4° eversion (reference 0.6° eversion). Average foot clearance height increased from 36.7 to 49.7 mm (reference 54.3 mm), and ankle angle in late swing changed from 6.2° of plantarflexion to 0.7° of dorsiflexion (reference 2.7° of dorsiflexion).

With the exoskeleton, minor restriction of maximum plantarflexion in preswing was observed; compared to the reference condition, the maximum plantarflexion angle in preswing with the exoskeleton (9.8°) was largely unchanged (reference 13.1°).

## 5 Discussion

In this study, we have designed and developed a new powered soft exoskeleton to assist people with dropfoot, with or without a tendency for excessive inversion. We tested the device's overall feasibility and efficacy in a small group of non-disabled adults, first in several seated tests that simulate problematic gait phases for persons with dropfoot, specifically foot clearance in swing and early foot contact in loading response, then during gait. As a proxy for the associated atypical motor control in persons with dropfoot, external plantarflexion and inversion moment were created in the non-disabled subjects by a weight on the superior, distal, and lateral side of the foot, inspired by previous studies that simulated gait deviations in non-disabled subjects (Hong et al., 2021; Zhang Q. et al., 2023). The purpose of the third test in our study was to evaluate the ability of the controller to compensate for the inherent resistance in the exoskeleton system.

The novelty of our device is its ability to control both the sagittal and frontal motion of the ankle, achieved with the relatively simple design of placing an anchor for the cable on the lateral and another on the medial sides of the forefoot. The assistance force and design with motors in the waist belt and Bowden cables were inspired from previous designs (Awad et al., 2017; Bae et al., 2018; Lerner et al., 2018), with the feature of being lightweight and compact. Our design was able to control forefoot kinematics with low errors, notably in the frontal plane, compared with a design described by Xia et al. (2020). Instead of employing a rigid structure on the foot segment (Zhong et al., 2023) or controlling the movements in the sagittal and frontal planes separately (Xia et al., 2020), our device uses two motors to lift the medial and lateral sides of the foot and thus adjust in both planes in a relatively simple but effective control strategy.

The two-mode controller in our design includes position control. The controller's accuracy of ankle kinematics is a common yet important performance metric to evaluate a device's effectiveness, particularly for people with disabilities (Yeung et al., 2017; Bae et al., 2018; Xia et al., 2020). In the first two tests, we found that the dorsiflexion and eversion assistance tracked the target position with relatively low errors, which demonstrates that our exoskeleton is capable of guiding the ankle motion well with the cable retraction/release mechanism.

Soft exoskeletons commonly have lower tracking accuracy than rigid exoskeletons (Asbeck et al., 2013; Bae et al., 2018), partly attributable to movement and/or deformation of the textiles and other soft materials subjected to interaction force. In our experiments, we did not observe any slippage of the calf wrap. It did, of course, deform, however, but this effect was mitigated to a good extent by setting the target ankle position and corresponding cable length before the experiment with the exoskeleton on. However, in Test 1, tracking errors were higher in the fast condition than in the slow condition, which might be due in part to more textile deformation with the larger interaction forces.

The second mode of the controller was the force-free controller. The simplicity of the design with both cable anchors on the forefoot also presents a challenge in assisting ankle motion as it introduces device resistance during plantarflexion. Bae et al. (2018) have suggested an approach to address this issue, specifically a cable slackness management method, which involved updating a baseline position and then releasing the cable to the position during each stride. For our implementation, this approach would not be appropriate, particularly as ankle kinematics may deviate in each stride, making it difficult to adjust cable tension for each step. We implemented the force-free controller, aiming at compensating for the inherent drag from the system friction and cable slack and making the exoskeleton follow natural ankle plantarflexion as smoothly as possible. The experimental result showed that the force-free controller was able to reduce the inherent drag of the system resistance, apparent as higher angle velocity in passive plantarflexion in Test 3, but not to eliminate it entirely.

In the gait tests, the exoskeleton showed potential in normalizing the altered ankle and foot kinematics induced by the simulated gait impairment; specifically, it was able to lift the foot during swing and orient the foot segment similarly to the reference condition, without limiting the plantarflexion motion during preswing. Switching between control modes involves a 3–5 ms delay, which is unlikely to have much influence on the device's efficacy. Further exploration of other control profiles and optimization methods for ideal assistance is warranted.

There are some limitations and simplifications in this study. In Test 3, the force-free controller could reduce some of the effects of drag in plantarflexion in both seating and gait tests but not entirely. In addition, it did not constrain the ankle from inverting. This could be problematic for persons with a tendency to invert the ankle; more tension in the lateral Bowden cable may be required. For the parameter *K*_*t*_ in the force-free controller, we selected a constant value to balance the flexibility and stability for all subjects in both seating and gait tests. Future investigations are necessary to determine the optimal value to provide adequate assistive and balance plantarflexion and inversion control. It is also possible that optimal values of *K*_*t*_ may be subject-specific, based on motor control and subject anthropometry. We did not examine the device on persons with dropfoot in the current study; the aim of the study was instead to propose the device and perform proof-of-concept tests prior to testing on a subject population with gait disability. By adding the weight over the foot to simulate dropfoot gait, we were able to capture several gait characteristics similar to those observed spontaneously in persons with dropfoot, though of course the two scenarios are not identical. Testing exoskeletons on subjects with simulated impairments is a common practice before their application, often involving the addition of resistance, such as springs (Xia et al., 2020; Hong et al., 2021), to specific joints or segments. These limitations will be addressed in future study involving individuals with a tendency for dropfoot and excessive ankle inversion.

## 6 Conclusion

In this study, a 2-DoF powered soft ankle exoskeleton was developed to assist the ankle in movements that simulate dropfoot and excessive inversion. The device's features are its low weight and minimal movement restriction. In the pilot study population of non-disabled persons, the exoskeleton was able to accurately guide the ankle in active dorsiflexion and eversion, as well as in controlled plantarflexion and inversion. The force-free control was able to compensate for a significant portion of the inherent device resistance, though not all. The exoskeleton showed promise in normalizing dropfoot-related ankle and foot kinematics in swing and initial contact while not restricting plantarflexion in preswing. Altogether, these demonstrated its feasibility for use to control foot and ankle kinematics, and its potential to counteract dropfoot with or without excessive inversion. Future study will address its efficacy during gait in a population with these gait deviations.

## Data Availability

The original contributions presented in the study are included in the article/supplementary material, further inquiries can be directed to the corresponding author.
